# Acupuncture improves the symptoms, serum ghrelin, and autonomic nervous system of patients with postprandial distress syndrome: a randomized controlled trial

**DOI:** 10.1186/s13020-024-01028-3

**Published:** 2024-11-20

**Authors:** Zi-tong Fu, Cun-zhi Liu, Mi-Rim Kim, Yi-duo Liu, Yu Wang, Yi-Ming Fu, Jing-Wen Yang, Na-Na Yang

**Affiliations:** International Acupuncture and Moxibustion Innovation Institute, School of Acupuncture-Moxibustion and Tuina, Beijing University of Chinese Medicine, No. 11 Bei San Huan Dong Lu, Chaoyang District, Beijing, 100029 China

**Keywords:** Postprandial distress syndrome, Acupuncture, Randomized controlled trial, Serum ghrelin, Autonomic nervous system

## Abstract

**Background:**

Whether gastrointestinal hormones in patients with postprandial distress syndrome (PDS) are altered by acupuncture, and whether such alterations are related to the autonomic nervous system (ANS), remains an open question.

**Objective:**

This study aims to investigate the effects of acupuncture on symptoms, serum hormones, and ANS in PDS patients.

**Methods:**

This randomized controlled clinical trial was conducted at Beijing Hospital of Traditional Chinese Medicine affiliated with Capital Medical University. Sixty-two PDS patients were randomly assigned equally to acupuncture or sham acupuncture arm (3 sessions per week for 4-week). The main outcome measures which were evaluated at baseline and 4-week included cardinal symptoms, serum hormones including ghrelin, vasoactive intestinal peptide (VIP), substance P (SP), and ANS.

**Results:**

Among the 62 randomly assigned participants, 51 (82%) were included in the baseline characteristics and outcome analysis. Gastrointestinal symptoms including response rate (p = 0.001) and dyspepsia symptom severity (p = 0.002) were significantly improved after acupuncture treatment. Serum ghrelin concentration was significantly higher in acupuncture group than in sham acupuncture group (8.34 ± 3.00 ng/ml versus 6.52 ± 2.00 ng/ml, p = 0.022) after 4-week treatment, instead of VIP and SP (p > 0.05). The acupuncture group had significantly higher vagal activity, showing with increasing of high-frequency component (HF, p ≤ 0.001) and decreasing of the ratio of low-frequency and HF (p ≤ 0.001). In relationship analysis, the HF component exhibited a significant inverse correlation with symptom severity (R = − 0.501, *p* ≤ 0.001), but not with ghrelin level (R = 0.026, *p* = 0.865).

**Conclusion:**

Acupuncture may improve the symptoms and increase the ghrelin level of PDS patients, the therapeutic effect of acupuncture was associated with the alteration of vagal activity.

*Trial registration*: The trial is registered with the ISRCTN registry, ISRCTN12511434. Registered 31 March 2017, https://www.isrctn.com/.

**Supplementary Information:**

The online version contains supplementary material available at 10.1186/s13020-024-01028-3.

## Background

Functional dyspepsia (FD) is a common functional gastrointestinal disorder with symptoms including upper abdominal discomfort or pain that affects up to 16% of the general population [1, 2]. Rome IV criteria divided FD into 2 subtypes: postprandial distress syndrome (PDS) with postprandial fullness and early satiation, and epigastric pain syndrome (EPS) with epigastric pain and burning [3]. As the most common subtype in FD, PDS was reported to lead excessive burden on health-related quality of life and economic expenditures [1, 4].

Although the pathophysiology of PDS is complex and not fully understood, growing evidence have suggested that the disorder of brain-gut dialogue can recognize as a major pathogenesis mechanism in PDS [5]. Particularly, FD was defined by Rome IV criteria as a disorder of brain-gut interaction. As a bidirectional link between the brain and the gastrointestinal tract, the autonomic nervous system (ANS) plays a crucial role in gastrointestinal homeostasis, such as motility, immunity, acid secretion, and enteric hormone release, through its sensing and modulatory roles [6, 7]. Restoring the balance of ANS may be a therapeutic target as low vagal tone and greater sympathetic drive were observed in a variety of gastrointestinal disorders.

Acupuncture has been practiced empirically in functional gastrointestinal disorders for several millennia, its efficacy was evaluated by many systemic reviews [8–10] and randomized controlled trials (RCTs) [11–13]. Moreover, our previous study found that symptoms of PDS patients could be alleviated by 12 weeks of acupuncture, and the improvement was maintained during a 12-week post-treatment follow-up [14]. Acupuncture is a promising treatment to alter ANS activity. Moreover, acupuncture is also believed to generate its therapeutic effects by the release of neuropeptides and modulation of the ANS balance in the animal model [15, 16]. However, it is unknown whether acupuncture is capable of concurrently improving ANS functions and gastrointestinal hormones in patients with PDS who have been reported to exhibit low vagal tone and neuropeptide disturbance. This placebo-controlled randomized clinical trial was performed to explore the efficacy of acupuncture on PDS while evaluating the alteration of hormone-secreting and ANS functionality assessed by heart rate variability (HRV).

## Methods

### Trial design

This study investigated the effects of acupuncture on symptoms, serum hormones, and ANS in PDS patients. Sixty-two patients were enrolled between October 2017 to October 2018 in China primarily based on a major randomized clinical trial [14]. This study was approved by the institutional review board and ethics committee of Beijing Hospital of Traditional Chinese Medicine affiliated with Capital Medical University and was conducted in accordance with the Consolidated Standards of Reporting Trials (CONSORT) reporting guideline [17]. All patients provided their written informed consent before participant. The study was registered at the Chinese Trial Registry (ISRCTN12511434).

### Participants

Patients aged 18–65 years (either sex) who met the Rome IV diagnostic criteria for PDS were recruited. A negative esophagogastroduodenoscopy within a year was needed for patients over 35 years old [18–20]. Patients who didn’t take dyspepsia drugs for a minimum of 2 weeks before enrollment, receive acupuncture treatment in the last month, or participate in any other trial in the previous 2 months were eligible. Major exclusion criteria included any serious diseases and prior surgery related to the gastrointestinal tract that induced dyspepsia symptoms (eTable 1).

### Randomization and blinding

Eligible patients were randomly allocated to the acupuncture group or sham acupuncture group in a 1:1 ratio. The randomization sequence was generated by an independent statistician with SAS, version 9.0 software (SAS Institute, Cary, North Carolina, USA), procedure PROC PLAN. The random number assignment was conducted by an independent administrator unaffiliated with the study. The random number was assigned after eligible patients completed baseline assessments. Patients were treated by appointment to maintain blinding and avoided communication with each other by an opaque curtain. The outcome evaluators and statisticians were also blinded to treatment assignment. Due to the nature of acupuncture, the acupuncturists were be blinded.

### Interventions

Study interventions program were developed by our previous studies [14, 21, 22] and expert consensus [23]. Treatments were performed by licensed acupuncturists who have at least a 5-year undergraduate education and a 3-year clinical experience. All acupuncturists received standardized operating procedure training before patient enrollment.

Eight basic acupoints and one of three optional acupoint were applied to the acupuncture group. The eight basic acupoints were bilateral *Neiguan* (PC6), *Tianshu* (ST25), *Zusanli* (ST36), *Gongsun* (SP4) and unilateral *Baihui* (DU20), *Danzhong* (RN17), *Zhongwan* (RN12), *Qihai* (RN6). The following optional acupoints were selected based on different types of TCM syndromes: *Taichong* (LR3) for depression of the *qi* of the liver; *Taibai* (SP3) for weakness of the *qi* of the spleen and stomach; and *Neiting* (ST44) for damp-heat in the stomach. After needle insertion, manipulation of twirling and rotating, lifting, and thrusting was done to achieve the *de qi* sensation at all acupoints. For the sham acupuncture group, six non-acupoints were inserted to a depth of 2–3 mm without *de qi*. The location of these acupoints and non-acupoints are shown in eFig 1 and eTable 2.

Participants in both groups started treatment on the day of randomization and received twelve 20-min sessions over 4 consecutive weeks (3 sessions per week). Sterile, disposable acupuncture needles (0.25*25 mm or 0.25*40 mm [Hwatuo]) were used in both groups.

### Outcomes

#### Primary outcome

Response rate based on overall treatment effect (OTE) was assessed at the end of treatment, using the self-assessment questionnaire of global symptom improvement, which has been used in numerous FD studies [14, 21, 22]. Patients were asked, “whether dyspepsia symptoms improved during the past week compared to the baseline period?” The answer was scored by a 7-point Likert scale, with options of “extremely improved”, “improved”, “slightly improved”, “not changed”, “slightly aggravated”, “aggravated”, or “extremely aggravated”. Patients who answered, “extremely improved” or “improved” were considered responders.

#### Secondary outcomes

1. Symptoms assessment
  Severity of dyspepsia symptom (postprandial fullness, early satiety, upper abdominal bloating, epigastric pain, epigastric burning, nausea, vomiting, and belching) were assessed by Symptom Index of Dyspepsia scale (SID) at baseline and the end of treatment [24]. SID was 4-point Likert scale with a ranging score of 0 to 4 for each item: asymptomatic (0 points); mild (1 points); moderate (2 points); severe (3 points). Higher scores indicate more severe dyspepsia symptoms.
2. Disease-specific quality of life
  Disease-specific quality of life was assessed by a 25-item Nepean Dyspepsia Index (NDI) [25] at baseline and the end of treatment. NDI measured the specific life quality of PDS patients in four domains, namely, interference (13 items), know/control (7 items), eat/drink (3 items), and sleep/disturb (2 items). Each item is scored by a 5-point Likert scale that ranges from “not at all” to “extremely”, with higher scores indicating a better quality of life.
3. Psychological status assessment
  The depression and anxiety status was evaluated by the Hospital Anxiety Depression Scale (HADS) [26] at baseline and the end of treatment. This self-administered scale consists of 14 items split across anxiety and depression subscales, each with a four-point ordinal response format with values ranging from 0 to 3, which resulted in scale values between 0 and 21 for each subscale. Higher scores indicate more severe symptoms.
4. ANS assessment
  To evaluate ANS function, HRV was tested by AR12 type electrocardiograph (Huntleigh Healthcare., Cardiff, UK, Leupamed GmbH, Graz, Austria) in PDS patients. Subjects were studied in a quiet room maintained at a comfortable temperature (22–24 ℃) between 8:00 am to 11:00 am. Removing mobile devices or other electronic equipment from the vicinity that may interfere with paper data collection. The patients could not do extreme physical exercises the day before, or take medicine/stimulant substances and beverages that can potentially change the HRV, and had a good night’s sleep. After an overnight fast, each subject was studied in the supine position after breathing smoothly for 10 min to achieve acclimatization with spontaneous breathing (~ 11–20 bpm), and then electrocardiography (ECG) recording was started for 30 min. During the data collection, the patients could not talk, sleep, sneeze, cough, or move. The HRV were performed *time-domain* and *frequency-domain* measurements. *Time-domain* components of HRV were analyzed and compared using a recording of equal length (30 min) for each subject, and the following parameters were identified: Standard deviation of NN intervals (SDNN) and the root mean square of successive RR interval differences (RMSSD, indicator of vagal nerve activity). In the *frequency-domain* of HRV, the following measures were collected: TP power (total power of the 0.0–0.4 Hz), HF power (relative power of the high-frequency 0.15–0.4 Hz in normal units), LF power (relative power of the low-frequency 0.04–0.15 Hz in normal units), LF/HF ration.
5. Hormones assessment in plasma
  Fasting blood was collected at baseline and the end of treatment. The blood samples were centrifuged to separate the plasma by 3000 r/min for 30 min (D-37520, Thermo Fisher, Germany) and then stored at – 80 ℃. Vasoactive intestinal peptide (VIP: EK-064-16, Phoenix Pharmaceuticals, USA), ghrelin (EK-031-30, Phoenix Pharmaceuticals, USA), and substance P (SP: ADI-900-018, Enzo Biochem, USA) level in plasma were measured by enzyme-linked immunosorbent assay (ELISA) according to the manufacturer’s protocol.

### Statistical analysis

As for sample size calculation, according to the previous results and literature studies, we estimated that the OTE-based response rates of the acupuncture group and the sham acupuncture group would be 70% and 30%, respectively. A minimum sample size of 25 patients in each group was estimated to have at least 90% power to detect a 2-sided significance level of 5% based on response rate. Considering the 20% shedding rate, 30 patients should be included in each group, for a total of 60 patients.

The measurement data were expressed by the mean ± standard deviation (SD) and counting data were expressed by cases (percentages). The response rate was compared using the Pearson Chi-square test. The secondary outcomes including SID, NDI, HADS, HRV, and hormones between groups were analyzed with the Student’s t-test for normality and Mann–Whitney U test for un-normality. The correlation analysis was detected by Spearman’s correlation analyses when the variables were distributed non-normally. For normally distributed variables, Pearson correlation analyses were conducted. The mediation analysis was detected by Bootstrap’s mediation analysis to study whether acupuncture can indirectly affect gastrointestinal hormone levels through changes in ANS. All statistical analyses were performed using SPSS 20.0 software with a 2-sided *p-value* of < 0.05 considered significant.

## Results

### Patient characteristics

**Fig. 1:**
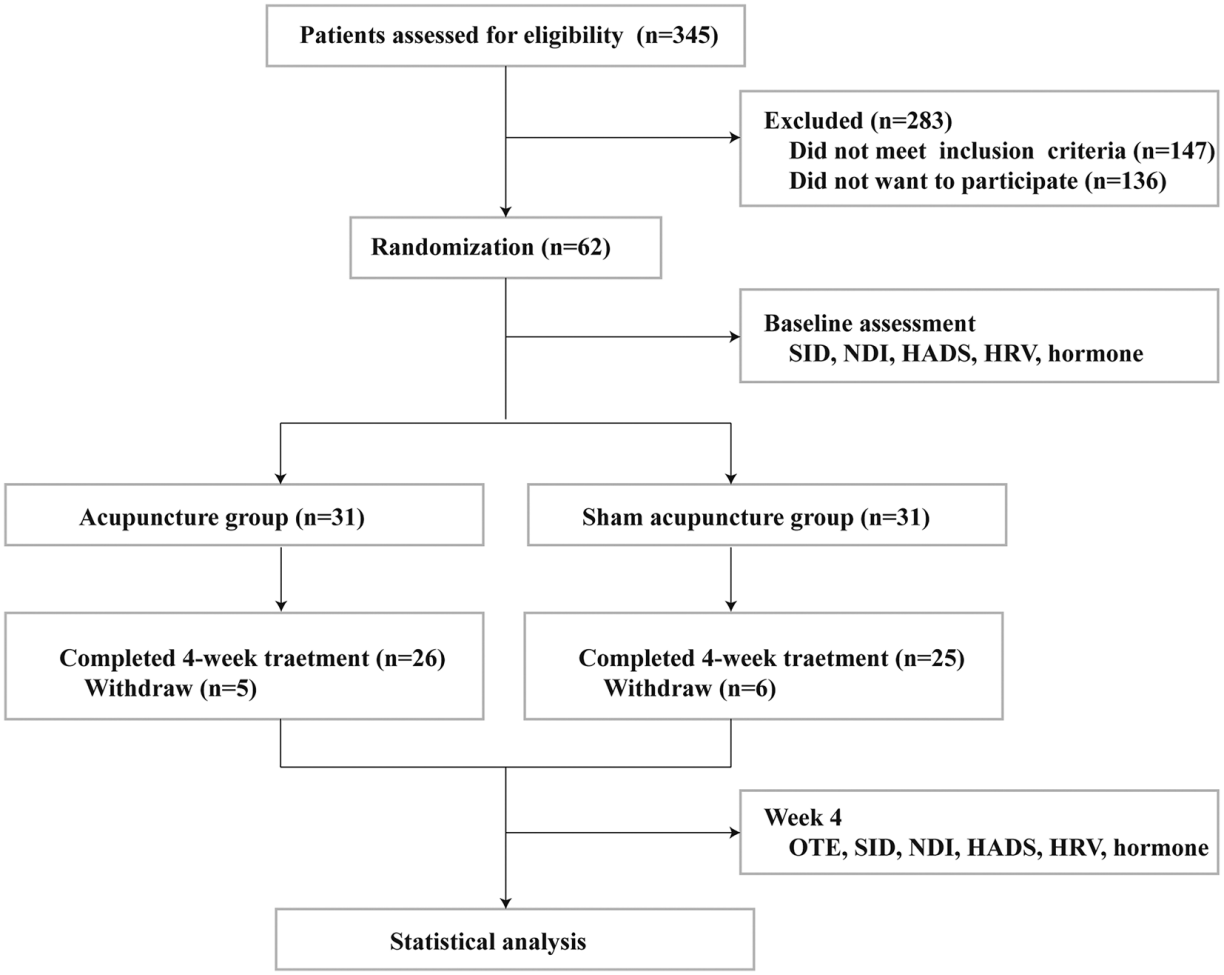
Trial flow diagram

Initially, a total of 345 consecutive participants with dyspepsia symptoms were evaluated and screened from October 2017 to October 2018; Of these, 283 participants (82%) were excluded (147 participants [42.6%] did not meet inclusion criteria; 136 participants [39.4%] did not want to participate (n = 136). Consequently, 62 of them were qualified for this study and randomly assigned to receive either acupuncture (n = 31) or sham acupuncture (n = 31). 11 participants withdrew from this study (5 in the acupuncture group and 6 in the sham acupuncture group) for the following reasons: inability to be contacted (n = 1), inability to complete treatment due to travel (n = 2), refusal to be tested (n = 5), or poor efficacy (n = 3). Consequently, 26 patients in the acupuncture group and 25 patients in the sham-acupuncture group were incorporated in the analysis of baseline characteristics and outcomes (Fig. 1). The baseline demographic and clinical characteristics were similar between groups (*p* > 0.05, Table 1).

**Table 1:** Baseline characteristics

	Acupuncture group (n = 26)	Sham acupuncture group (n = 25)
Sex, n (%)		
Female	20 (76.9%)	17 (68.0%)
Male	6 (23.1%)	8 (32.0%)
Age, in years	42.88 ± 16.75	41.28 ± 15.69
Course of disease, mo	42.69 ± 54.07	59.92 ± 65.88
BMI, kg/m^2^	22.45 ± 2.98	23.42 ± 3.26
SID	7.35 ± 2.87	8.04 ± 2.49
Postprandial fullness	1.73 ± 0.72	2.00 ± 0.65
Early satiety	1.04 ± 0.82	1.12 ± 0.97
Upper abdominal bloating	1.58 ± 0.70	1.60 ± 0.71
Epigastric pain	0.46 ± 0.71	0.64 ± 0.81
Epigastric burning	054 ± 0.71	0.72 ± 0.84
Nausea	0.54 ± 0.71	0.48 ± 0.71
Vomiting	0.23 ± 0.51	0.20 ± 0.65
Belching	1.23 ± 1.03	1.16 ± 1.03
NDI	78.83 ± 2.56	78.18 ± 3.02
HADS	10.54 ± 5.11	8.16 ± 6.06

*BMI* body mass index, *SID* symptom index of dyspepsia scale, *NDI* nepean dyspepsia index, *HADS* hospital anxiety depression scale

### Primary outcome

The "extremely improved" and "improved" responders were 11 (42.3%) and 9 (34.6%) in the acupuncture group, and 2 (8%) and 5 (20%) in the sham acupuncture group, separately. Therefore, the response rate based on OTE at the end of treatment was 20 (76.9%) in patients with acupuncture treatment versus 7 (28%) in patients with sham acupuncture treatment (*p* = 0.001, Table 2).

**Table 2:** Treatment outcomes of patients with postprandial distress syndrome

	Acupuncture group (n = 26)	Sham acupuncture group (n = 25)	*p* value^a^
OTE, n (%)	20 (76.9%)	7 (28%)	0.001^*^
Change from baseline in SID	− 4.46 ± 2.30	− 2.28 ± 2.44	**0.002**^**c**^
Postprandial fullness	− 1.0 [− 2.0, 0.0]	0.0 [− 1.0, 0.0]	**0.017**^b^
Early satiety	− 1.0 [− 1.0, 0.0]	0.0 [0.0, 0.0]	**0.020**^b^
Upper abdominal bloating	− 1.0 [− 2.0, − 0.8]	0.0 [− 1.0, 0.0]	**0.005**^b^
Epigastric pain	0.0 [− 1.0, 0.0]	0.0 [− 0.5, 0.0]	0.650^b^
Epigastric burning	0.0 [− 1.0, 0.0]	0.0 [− 1.0, 0.0]	0.874^b^
Nausea	0.0 [− 1.0, 0.0]	0.0 [− 1.0, 0.0]	0.974^b^
Vomiting	0.0 [0.0, 0.0]	0.0 [0.0, 0.0]	0.185^b^
Belching	− 1.0 [− 1.0, 0.0]	0.0 [− 1.0, 0.0]	0.165^b^
Change from baseline in NDI	− 10.0 [− 25.50, − 1.75]	− 4.0 [− 13.5, 0.0]	0.124^b^
Change from baseline in HADS	− 1.5 [− 4.0, 0.0]	− 1.0 [− 2.0, 1.0]	0.109^b^

Values are reported as mean ± SD unless otherwise indicated, *P-*values in bold represent significant differences

*OTE* overall treatment effect, *SID* symptom index of dyspepsia scale, *NDI* nepean dyspepsia index, *HADS* hospital anxiety depression scale

^a^All tests were two-sided. *p* < 0.05 was considered significant

^b^Calculated using Mann–Whitney U test

^c^Calculated using Student t-test

^*^Calculated using Pearson Chi-aquare test

### Secondary symptom outcomes

The average change from baseline in dyspepsia symptom severity was 4.46 (95% CI 3.53–5.39) in the acupuncture group and 2.28 (95% CI 1.27–3.29) in the sham acupuncture group at the end of treatment (*p* = 0.002). For the individual three core symptom scores of SID, including postprandial fullness (*p* = 0.017), early satiation (*p* = 0.020), and upper abdominal bloating (*p* = 0.005), were significantly decreased in the acupuncture group compared with sham acupuncture group after interventions. No differential treatment effects were seen for epigastric pain, epigastric burning, nausea, vomiting, and belching (*p* > 0.05, Table 2). Meanwhile, there were no statistically significant differences between groups in the average change from baseline in the NDI (*p* = 0.124) and HADS (*p* = 0.109) scores (Table 2). Nine (34.6%) adverse events occurred in the acupuncture group, and seven (28%) occurred in the sham acupuncture group (eTable 3). No serious adverse events were reported in either group.

### Hormones, HRV, and correlation analysis

**Fig. 2:**
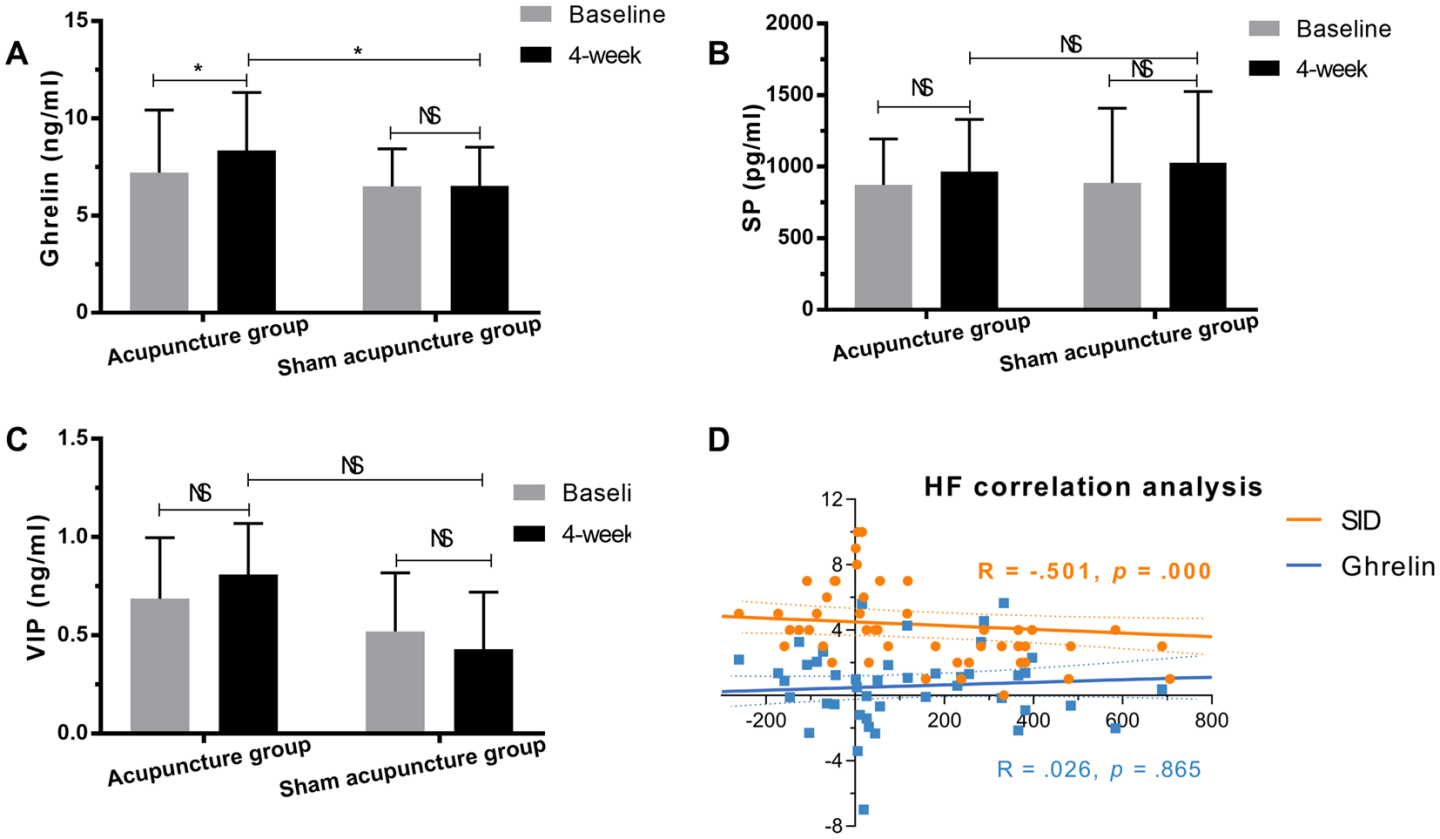
Effects of acupuncture on hormones and the correlation between vagal nerve, ghrelin, and symptoms. Level of plasma ghrelin (**A**), SP (**B**), and VIP (**C**). The relationship between the changing of HF from baseline and SID changed scores or plasma gremlin (**D**) using Spearman’s correlation. Solid lines represent the regression lines (predictor for a given X-axis value), and broken lines are the 95% confidence limits for the regression lines. *VIP* Vasoactive intestinal peptide, *SP* substance P, *SID* symptom index of dyspepsia scale, *HF* high-frequency component, *NS* no significant difference; *p < .05

To explore possible effect mechanism mechanisms for these dyspepsia symptoms, plasma ghrelin, SP, and VIP levels were evaluated at the baseline and the end of treatment (Fig. 2 and eTable 4). The mean amount of plasma ghrelin measured by the ELISA was 7.21 ± 3.23 ng/ml at baseline 8.34 ± 3.00 ng/ml at week 4 in the acupuncture group. 6.49 ± 1.94 ng/ml at baseline, 6.52 ± 2.00 ng/ml at week 4 in the sham acupuncture group. The increase of ghrelin at week 4 was greater in the acupuncture group than in the sham acupuncture group (*p* = 0.022, Fig. 2A). However, no statistically difference was found in plasma VIP and SP at week 4 (*p* > 0.05, Fig. 2B, C and eTable 4).

In *time-domain indices*, RMSSD was markedly increased in the acupuncture group compared with the sham acupuncture group (*p* < 0.0001) after 4-week treatment, but not SDNN (*p* = 0.258). In frequency-domain measurements, HF was also higher in the patients who underwent acupuncture treatment (*p* = 0.000). Meanwhile, LF/HF ratio in the acupuncture group was decreased compared to the sham acupuncture group (*p* = 0.000). However, no difference was found between the two-groups in LF and TP at week 4 (*p* > 0.05, Table 3). These results indicated that acupuncture had the potential to ameliorate the disorder of ANS, mainly showing the activating of the vagal nerve.

**Table 3:** Effects of acupuncture on autonomic function

	Acupuncture group (n = 26)	Sham acupuncture group (n = 25)	*p* value^a^
SDNN	0.47 [− 5.43, 7.73]	− 4.46 [− 7.42, 6.35]	0.258
RMSSD	3.95 [1.98, 9.78]	− 2.53 [− 8.77, 2.96]	**0.001**
LF	32.55 [− 148.75, 190.73]	− 48.00 [− 209.70, 210.65]	0.451
HF	331.10 [174.43, 480.28]	− 44.40 [− 106.25, 25.60]	**0.000**
LF/HF ratio	− 0.55 [− 0.99, − 0.07]	0.02 [− 0.19, 0.28]	**0.000**
TP	251.35 [− 244.43, 568.65]	− 133.90 [− 808.70, 506.90]	0.090

Values are reported as mean ± SD unless otherwise indicated, *P-*values in bold represent significant differences

*SDNN* the SD of beat-to-beat intervals, *RMSSD* the root mean square of successive differences, *LF* low-frequency component, *HF* high-frequency component, *TP* total power

^a^All tests were two-sided. *p* < 0.05 was considered significant. Calculated using Mann–Whitney U test

Furthermore, spearman’s correlation analysis was used to determine the correlation of HF components with SID scores and ghrelin levels. We found that the change in the HF component exhibited a significant inverse correlation with the changes in SID scores (R = − 0.501, *p* = 0.000), but not with ghrelin level (R = 0.026, *p* = 0.865, Fig. 2D). The mediation analysis was conduct with the change of HF as the mediating variable. We found that the direct effects of grouping on ghrelin were no significant (*β*(c’) = − 0.430877, *p* = 0.2098, *95% ci* [− 2.6167, 0.5913]).

## Discussion

This study showed that, compared with sham acupuncture, 12 sessions of acupuncture over 4 weeks provided impressively clinical relief of symptoms in PDS patients. A 48.9% higher response rate was noted for PDS patients with acupuncture versus sham acupuncture. Acupuncture also improved individual dyspepsia symptoms, especially in 3 cardinal symptoms (postprandial fullness, upper abdominal bloating, and early satiation). Meanwhile, acupuncture had the potential to increase plasma ghrelin levels and activate the vagal nerve, and the changes of vagal function were positively correlated with the improvements of symptoms severity. This result may support our hypothesis that acupuncture has a multimodal effect (including activating the vagal nerve and increasing gastrointestinal hormones) to improve PDS.

Concomitant with the increasing use of acupuncture, the efficacy of acupuncture on FD has been proved by several RCTs [14], although the potential mechanism is complex, multifactorial, and far from completely elucidated. Response rate based on OTE, as a subjective global evaluation, is meaningful in a clinical study for PDS. In previous trials of PDS, the response rate in the acupuncture group ranged from 61.8% points to 83% points [14, 21], which the response rate at acupuncture group (76.9%) of this study within this range. Besides, PDS is characterized by the presence of prevalently meal-related early satiation and fullness, so the actual symptom severity is as important as the perception of the disease. The previous study used the complete elimination rate of the three core symptoms (postprandial fullness, early satiation, and upper abdominal bloating) as the primary outcome, and found that acupuncture had the potential to improve core symptoms of PDS at 3 sessions per week [14, 22]. Similarly, the score of dyspepsia symptom severity in the acupuncture group of our trial was also significantly lower than the sham acupuncture group, especially in three core symptoms, indicating significant improvement in dyspepsia symptoms accompanied by better overall relief.

While the molecular mechanisms underlying FD remain poorly understood, it is thought that ANS imbalance and dysregulated gastrointestinal hormones are critical contributing factors. To study the possible involvement of certain neuropeptides, we assessed plasma “braking” hormones VIP and “accelerating” hormones, including ghrelin and SP at the baseline and the end of the treatment. Of interest, we demonstrated that acupuncture treatment significantly increased the level of ghrelin compared to sham acupuncture treatment in PDS patients, but not altering VIP or SP secreting. Ghrelin, a 28 amino acid motilin-related peptide with strong growth hormone-releasing activity, was shown to enhance gastric emptying and gastrointestinal motility [27–29]. In patients with idiopathic gastroparesis, administration of ghrelin enhanced gastric emptying and alleviated the meal-related dyspepsia symptoms [30]. Concomitantly, plasma ghrelin was evaluated in a study of acotiamide for FD patients, which revealed that pharmaceutical intervention significantly increased ghrelin secreting and improved impaired gastric emptying [31]. Against this backdrop, our results may suggest that improvement of PDS symptoms with acupuncture treatment could be associated with the upregulation of ghrelin levels.

Although the gastrointestinal tract possessed intrinsic neural plexuses that allowed a significant degree of autonomy over gastric functions, the central nervous system provided extrinsic neural inputs to control its functions via ANS [32]. The physiological functions of the vagal nerve largely overlap with the pathophysiological changes observed in FD [33], indicating that vagal dysfunction played a role in the development of disturbed gastric motility and perception [34] and it would be more informative to delineate the role of vagal nerve on FD. A wealth of clinical data suggested FD patients have impaired vagal efferent activity [35–37] which could be a therapeutic target. Surveys such as those conducted by *Lunding* [38] have shown that sham feeding was able to normalize meal-induced antral dysmotility in FD patients via up-regulate vagal tone. The FD patients showed decreased gastric accommodation and vagal activity assessing by the electrogastrogram compared with the healthy subjects, whereas, the transcutaneous auricular vagal nerve stimulation has therapeutic potential for FD patients by improving gastric accommodation and gastric pace-making activity via enhancing nerve activity [39].

HRV, a non-invasive and simple method for the quantitative evaluation of autonomic activity, is used as an assessment method for cardiac autonomic modulation [40]. Although there is no direct assessment of gastroduodenal vagal efferent activity in FD patients, the gastroduodenal vagal efferent shares the same central origin with the cardiac vagal efferent [41]. Therefore, HRV was used to evaluate the effect of acupuncture on sympathetic-vagal autonomic balance in patients with PDS [42, 43]. Here, we found that TP, the sum of the energy in the LF and HF bands, was similar in patients at week 4, indicating that it was able to directly compare the *frequency-domain* measurements of the two groups. The HF and RMSSD as indicators of vagal nerve activity were significantly increased after 4-week acupuncture treatment. Additionally, sympathetic activity was primarily associated with the LF, which was similar in the two groups. Meanwhile, the LF band power makes a significant contribution to SDNN [40] which was also similar in different groups indicating that acupuncture stimulation could alter vagal nerve activity in PDS patients. More importantly, LF/HF ratio was to estimate the sympathetic-vagal autonomic balance. We also found that this ratio was lower after 4-week acupuncture treatment suggesting an enhanced parasympathetic outflow. This result is in agreement with others reported by *Hausken* [44] and *Undeland* [45]. Therefore, Acupuncture may achieve therapeutic effects on PDS by activating the vagal nerve.

Vagal nerve exerts a predominantly excitatory effect upon gastrointestinal muscle. The stomach receives an especially dense vagal innervation which gradually decreases distally [32]. This was consistent with our finding, in which vagal nerve excitement was negative with the severity of dyspepsia symptoms. Other studies showed that the vagal nerve altered the production of gastrointestinal hormones, but not the sympathetic nerve [46–48]. In this trial, we found that acupuncture was able to increase vagal tone, enhance ghrelin levels, and improve dyspepsia symptoms. Another assumption that cannot be ruled out is that the effect of acupuncture on the increased ghrelin secretion is a consequence of vagal nerve excitement in PDS patients. However, correlation analysis showed that the vagal nerve was not related to ghrelin level. Previous studies found that ghrelin is known to inhibit vagal afferent responses, therefore a reduction in ghrelin levels is likely to lead to an increase in sensitivity of vagal afferent which contribute to the early satiety or bloating [33, 49]. Reciprocally, acupuncture stimulation had potential to increase vagal efferent activity, but not vagal afferent [50, 51]. Against this background, we found that ANS balance and hormones were independent targets in acupuncture treatment, and there was no causal relationship between these two targets.

The present study has several limitations. First, the primary outcome in this trial was subjective and vulnerable to recall bias. Nonetheless, OTE still is one of the commonly used primary outcomes due to no universally accepted endpoint established in the FD trial [52, 53]. Second, the ANS activity and plasma hormones were only evaluated at the end of the 4-week treatment; the long-term effect of acupuncture on these indices could not be determined. Our previous study found that the therapeutic effect was sustained over 12 weeks in PDS patients who received thrice-weekly acupuncture for 4 weeks compared with sham acupuncture treatment [14]. However, it remains unknown that whether the long-term effect of acupuncture was also mediated by increasing the vagal nerve activity and ghrelin level or not. Third, acupuncturists could not be blinded, and blinding assessment of patients was not performed in our trial. Finally, the dropout rate of the sample was higher than expected, which may compromise the results of this study. The main contributor to this 17.7% was younger patients who have heavier work and less free time.

## Conclusion

Acupuncture treatment can improve clinical symptoms and increase patient-reported adequate relief rate compared with the sham acupuncture in PDS patients. The therapeutic effects of acupuncture are associated with increasing vagal nerve activity and enhancing plasma ghrelin levels, rather than SP and VIP. To sum up, this study provided evidence that acupuncture treatment was efficacious for PDS and its key individual symptoms via altering ANS function and increasing plasma ghrelin levels. Additionally, larger and multicenter study is warranted to evaluate a longer-term treatment and follow-up.

## Supplementary Information

Supplementary Material 1.

## Data Availability

The datasets used and/or analysed during the current study are available from the corresponding author on reasonable request.

## Abbreviations

FD: Functional dyspepsia
PDS: Postprandial distress syndrome
EPS: Epigastric pain syndrome
ANS: Autonomic nervous system
HRV: Heart rate variability
CONSORT: The Consolidated Standards of Reporting Trials
OTE: Overall treatment effect
SID: Symptom Index of Dyspepsia scale
NDI: Nepean Dyspepsia Index
HADS: Hospital Anxiety Depression Scale
ECG: Electrocardiography
SDNN: Standard deviation of NN intervals
RMSSD: Root mean square of successive RR interval differences
VIP: Vasoactive intestinal peptide
SP: Substance P
ELISA: Enzyme-linked immunosorbent assay
SD: Standard deviation
