# Supplementary material for: Acupuncture improves the symptoms, serum ghrelin, and autonomic nervous system of patients with postprandial distress syndrome: a randomized controlled trial
Source: Chin Med. 2024 Nov 20;19:162. doi: 10.1186/s13020-024-01028-3 (PMC11580632; doi:10.1186/s13020-024-01028-3)
Supplement: Supplementary file 1 — Supplementary Material 1. [file 13020_2024_1028_MOESM1_ESM.docx]

**eTable 1. Inclusion and exclusion criteria for patients**

| **Patients with PDS** |
| --- |
| **Inclusion criteria**  1. age 15-65 years;  2. one or more of the following symptoms with 3 or more mean weekly for more than 3 months: postprandial fullness, upper abdominal. bloating or early satiation;  3. patients > 35 years need to provide normal esophagogastroduodenoscopy results within a year;  4. no acupuncture treatment in the last month;  5. not participated in any other trial in the previous 2 months.  **Exclusion criteria**  1. chronic and malignant disorders that might give rise to dyspeptic manifestations, such as diabetes mellitus, chronic liver disease, parasitic infestation, chronic pancreatic disorder, chronic renal failure, thyroid dysregulation, gastroparesis, gastrointestinal neoplasms, heart failure, etc;  2. signs of irritable bowel syndrome;  3. gastrointestinal tract associated surgery;  4. taking dyspepsia drugs, such as anti-secretary drugs, antacids, prokinetics, non-steroidal anti-inflammatory drugs, and antidepressant drugs within 2 weeks prior to enrollment;  5. difficulties in attending the trial, such as serious mental and physiological illness, dementia, or illiteracy;  6. severe coagulopathy;  7. drug or alcohol abuse;  8. pregnancy or lactating women. |

**eTable 2. Locations of acupoints and non-acupoints**

| **Acupoint** | **Location** |
| --- | --- |
| PC6 | Between the tendons of palmaris longus and flexor carpi radialis, 2 cun above the transverse crease of the wrist |
| ST25 | On the same level of the umbilicus and 2 cun lateral to the anterior midline |
| ST36 | 3 cun directly below patellar, and one figure-breadth lateral to the anterior border of the tibia |
| SP4 | On the medial aspect of the foot, anteroinferior to the base of the first metatarsal bone, at the border between the red and white flesh |
| DU20 | On the midline of the head, 7 cun* directly above the midpoint of the posterior hairline |
| RN17 | On the anterior midline, on the level of the 4th intercostal space, at the midpoint of the line joining the two nipples |
| RN12 | On the anterior midline, 4 cun above the unbilicus |
| RN6 | On the anterior midline, 1.5 cun below the umbilicus |
| LR3 | In the depression anterior to the junction of first and second metatarsal bones |
| SP3 | On the medial aspect of the foot, in the depression proximal to the first metatarsophalangeal joint, at the border between the red and white flesh |
| ST44 | On the dorsum of the foot, between the second and third toes, posterior to the web margin, at the border between the red and white flesh |
| Non-acu 1 | In the middle of Touwei (ST8) and Yuyao (EX-HN4) points |
| Non-acu 2 | 2 cun above the anterior superior iliac spine |
| Non-acu 3 | 2 cun below the umbilicus, and 1 cun lateral to the anterior midline |
| Non-acu 4 | In the middle of the medial epicondyle of the humerus and the styloid  process of ulna |
| Non-acu 5 | 3 cun below Yanglingquan (GB34), between the gallbladder and bladder meridian |
| Non-acu 6 | In the middle of Qiuxu (GB40) and Jiexi (ST41) points |

*1 cun (≈20 mm) is defined as the width of the interphalangeal joint of patient’s thumb. PC6, ST25, ST36, SP4, DU20, RN17, RN12, and RN6 were eight obligatory acupoints; LR3, SP3, and ST44 were optional acupoints; Others were non-acupoints.

**eTable 3. Adverse events**

|  | **Acupuncture group**  **(n=26)** | **Sham acupuncture group (n=25)** |
| --- | --- | --- |
| Serious adverse events | 0 | 0 |
| Subcutaneous hematoma | 9 (34.6%) | 7 (28.0%) |

Adverse events were counted to frequency in all participants.

**eTable 4. Level of plasma VIP, SP, and Ghrelin** (mean ± SD)

|  | **Acupuncture group** | | | **Sham acupuncture group** | | |  |
| --- | --- | --- | --- | --- | --- | --- | --- |
|  | Baseline | 4-week | *p_1_ value* | Baseline | 4-week | *p_2_ value* | *p_3_ value* |
| VIP (ng/ml) | 0.69 ± 0.33 | 0.81 ± 0.28 | .138 | 0.52 ± 0.30 | 0.78 ± 0.64 | .063 | .820 |
| SP (pg/ml) | 873.26 ± 321.26 | 967.19 ± 363.17 | .179 | 885.40 ± 524.44 | 1029.0 5± 101.25 | .134 | .615 |
| Ghrelin (ng/ml) | 7.21 ±3.23 | 8.34 ± 3.00 | **.041** | 6.49 ± 1.94 | 6.52 ± 2.00 | .946 | **.022** |

VIP: Vasoactive intestinal peptide; SP: substance P;

All tests were two-sided. *p* < .05 was considered significant.

*p_1_* and *p_2_*: Calculated using paired sample t test when compared between group.

*p_3_*: Calculated using independent-sample t test when compared between different groups at the end of treatment.


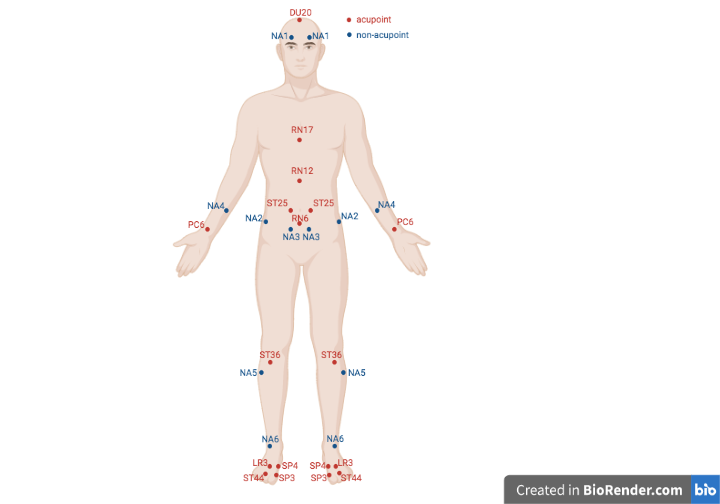


**eFig 1. Locations of acupoints and non-acupoints.** The red point represents acupoint, the blue point represents non-acupoint. NA, non-acupoint
